# Evolution in Laryngeal Cancer Mortality at the National and Subnational Level in Romania with 2030 Forecast

**DOI:** 10.3390/medicina61101743

**Published:** 2025-09-25

**Authors:** Andreea-Mihaela Banța, Nicolae-Constantin Balica, Simona Pîrvu, Karina-Cristina Marin, Kristine Guran, Ingrid-Denisa Barcan, Cristian-Ion Moț, Bogdan Hîrtie, Victor Banța, Delia Ioana Horhat

**Affiliations:** 1 Doctoral School, “Victor Babes” University of Medicine and Pharmacy Timisoara, 300041 Timisoara, Romania; andreea.banta@umft.ro (A.-M.B.); guran.kristine@umft.ro (K.G.); bogdan.hirtie@umft.ro (B.H.); 2 Department of Ear Nose Throat, “Victor Babes” University of Medicine and Pharmacy Timisoara, 300041 Timisoara, Romania; marin.karina@umft.ro (K.-C.M.); mot.ion@umft.ro (C.-I.M.); horhat.ioana@umft.ro (D.I.H.); 3 Institute of Public Health, Faculty of Medicine, University of Medicine and Pharmacy “Carol Davila” Bucharest, 020021 Bucharest, Romania; simona.parvu@umfcd.ro; 4 Governement of Romania, Ministry of Agriculture and Rural Development (MADR), 011791 Bucharest, Romania; victor.banta@madr.ro

**Keywords:** laryngeal neoplasms/mortality, Romania/epidemiology, forecasting, time series studies, geographic information systems, rural health, smoking/adverse effects

## Abstract

*Background and Objectives*: Laryngeal cancer imposes a disproportionate burden on speech, airway protection and long-term quality of life. Contemporary population-based data for Central and Eastern Europe remain scarce, and the post-pandemic trajectory is uncertain. *Materials and Methods*: We performed a nationwide, retrospective ecological time-series study using Romanian mortality registers and hospital-discharge files for 2017–2023. Crude and age-standardised mortality rates (ASMRs) were calculated, county-level indirect standardisation and spatial autocorrelation assessed and joinpoint regression quantified temporal trends. Forecasts to 2040 combined Holt–Winters/ARIMA models with Elliott-wave heuristics anchored to Fibonacci retracements. *Results*: In 2023, 798 laryngeal cancer deaths yielded a crude mortality of 3.65/100,000 (95% CI 3.41–3.91). Male mortality (7.07/100,000) exceeded female mortality 18-fold. Rural residents experienced a higher rate than urban counterparts (4.26 vs. 3.04/100,000), a difference unchanged after indirect age standardisation. National ASMR fell by 3.7% annually (*p* < 0.01), yet five counties formed a high-risk corridor (standardised mortality ratios 1.59–1.82); Moran’s I = 0.27 (*p* < 0.01) indicated significant spatial clustering. Pandemic-era surgical throughput collapsed by 48%, generating a backlog projected to persist beyond 2030. Ensemble forecasting anticipates a doubling of discharges and mortality between 2034 and 2037 unless smoking prevalence falls by ≥30% and radon exposure is curtailed. *Conclusions*: Although overall laryngeal cancer mortality in Romania is declining, the pace lags behind Western Europe and is threatened by geographic inequities and pandemic-related care delays. Aggressive tobacco control, radon-remediation policies and expansion of surgical and radiotherapeutic capacity are required to avert a forecasted surge in the next decade.

## 1. Introduction

Laryngeal cancer remains a relatively uncommon malignancy worldwide, yet its public health footprint is disproportionate because of the profound effects on speech, airway protection and long-term quality of life. According to the most recent GLOBOCAN 2022 update, the disease now ranks 20th for incidence (≈189,000 new cases) and 18th for mortality (≈103,000 deaths) globally, with Europe accounting for more than one-fifth of the burden [1]. Within head-and-neck malignancies, LC accounts for roughly 20–30% worldwide (≈185,000 of ≈890,000 HNSCC cases), with regional clinical series reporting up to 30–40% depending on population structure and risk profiles [1].

During the past three decades the age-standardised mortality rate (ASMR) for laryngeal cancer has fallen in many regions, yet the absolute number of deaths continues to climb as populations grow and age. A Global Burden of Disease (GBD) analysis covering 1990–2019 predicted that, without additional preventive action, worldwide deaths could rise a further 20% by 2035 [2]. Detailed GBD modelling also shows pronounced geographic heterogeneity: while Western Europe is experiencing a steady decline, several Central and Eastern European countries are trending upward or plateauing, largely reflecting divergent smoking trajectories and socioeconomic factors [3].

Tobacco and alcohol continue to drive most incident cases: combined, they explain over 80% of the global laryngeal cancer DALY burden [4,5]. Emerging evidence further implicates oncogenic human papillomavirus (HPV), especially genotypes 16 and 33, in a subset of supraglottic tumours, with a pooled HPV prevalence of 26% in recent meta-analyses [5]. Meta-analyses suggest that HPV DNA can be detected in a subset of laryngeal SCC and may be associated with modest survival benefits, though prevalence is considerably lower than in oropharyngeal cancer and varies by assay and geography [6].

Attention has also shifted toward occupational and environmental hazards. A 2024 meta-analysis confirmed a modest but significant excess risk among workers chronically exposed to wood dust (adjusted OR ≈ 1.14) [7]. Historical uranium miner cohorts first suggested a radon-linked excess of laryngeal cancer mortality [8], and the latest reviews underscore the need to integrate radon mitigation into head-and-neck cancer prevention strategies, particularly in high-exposure dwellings [9].

Pathologically, non-conventional variants such as laryngeal verrucous carcinoma exhibit distinct behaviour, with superior disease-specific survival compared with classic squamous counterparts, reinforcing the importance of histologic subtyping in prognostication [10].

Romania illustrates many of these global themes. National hospital data show that smoking prevalence among adult men remains above 30%, and a 2023 tertiary centre series from Bucharest reported that 88% of locally advanced cases presented with T3–T4 disease, reflecting late diagnosis and limited screening infrastructure [11].

The COVID-19 pandemic has further complicated the landscape. Multicentre studies from North America and Asia documented delayed presentations, stage migration toward more advanced tumours and longer time-to-treatment intervals during 2020–2021 [12,13]. Romanian surgical services were likewise disrupted; a 2024 analysis from Cluj-Napoca showed a 28% fall in head-and-neck oncologic case volume and an accompanying rise in tumour size at excision [14].

These converging trends underscore the need for robust national surveillance and forward-looking modelling. Contemporary guidelines now advocate risk-stratified imaging and biomarker follow-up to detect treatable recurrences earlier and allocate resources efficiently [15]. Building on these insights, the present ecological analysis interrogates Romanian mortality registers from 2017 to 2023, evaluates county-level inequities and applies Fibonacci–Elliott forecasting to project the post-pandemic trajectory through 2040, with the ultimate goal of informing targeted prevention and capacity-building strategies.

Quantifying Romania’s national and county-level mortality trends and spatial inequities is essential for targeting tobacco and radon control, optimising surgical and radiotherapy capacity and monitoring post-pandemic recovery. Forward-looking forecasts further enable proactive resource allocation and evaluation of policy scenarios (e.g., smoking reductions, radon remediation).

## 2. Materials and Methods

### 2.1. Study Design, Data Sources and Ethical Oversight

The present investigation was conceived as a nationwide, retrospective ecological time-series analysis that adheres to STROBE guidelines for observational research. The protocol received clearance from the Ethics Committee of the “Victor Babeș” University of Medicine and Pharmacy, Timișoara (IRB #615/14 February 2024). Because this was only anonymised, aggregate statistics were handled and the requirement for written informed consent was waived under EU GDPR 2016/679 and Romanian Law 190/2018. Mortality files that listed International Classification of Diseases, Tenth Revision (ICD-10) codes C32.0 through C32.9 as the underlying cause and hospital discharge records bearing the same codes were extracted from the Romanian National Institute of Public Health for each calendar year between 2017 and 2023. Denominators for rate calculations were derived from the National Institute of Statistics, which supplies mid-year resident population estimates stratified by age, sex, county, and settlement type. Linkage between the two national registries used the official county identification codes that remained unchanged throughout the study period.

This study addressed population-level surveillance questions (temporal trends, spatial clustering, and medium-term forecasting) using anonymised national aggregates. Individual exposures (e.g., smoking, alcohol, HPV status, radon) are not captured in the underlying registries; therefore, an ecological framework is appropriate for quantifying geographic and temporal patterns to inform public health and capacity planning.

### 2.2. Case Definition, Variable Set and Data Cleaning

A laryngeal cancer death was defined as any registry entry that recorded a C32.x code as the primary cause of death, whereas a laryngeal cancer discharge was any inpatient episode, live or fatal, whose principal diagnosis carried the same set of codes. Key analytic variables comprised age at event, sex, county of domicile, urban or rural residence and calendar year. Integrity checks excluded records with biologically implausible ages below twenty years, rows lacking a county identifier and duplicate episode numbers; altogether these exclusions affected fewer than 0.4 percent of entries. Age was subsequently regrouped into eleven five-year bands starting with 20–24 years and ending with 85 years or more. We excluded records with ages <20 years because LC mortality before early adulthood is exceedingly rare and yields unstable age-specific rates in small strata.

### 2.3. Rate Calculations and Age Standardisation

For every year and county, we calculated crude mortality rates by dividing the number of laryngeal cancer deaths by the corresponding population and multiplying by 100,000. Age-specific mortality rates were obtained for each five-year stratum. Direct age standardisation employed the Romanian resident population structure recorded in 2017, and gamma-distributed ninety-five-percent confidence intervals accompanied every directly standardised rate. Indirectly standardised mortality ratios were produced by applying national age-specific rates to county age structures and comparing observed with expected deaths. Finally, proportional mortality ratios were generated by expressing laryngeal cancer deaths as a percentage of all-cause deaths.

Indirect standardisation was applied at the county level, with expected deaths (Ec) calculated by multiplying national age-specific mortality rates (ra) by the corresponding county age-banded populations (Nc,a) and summing across age groups. The standardised mortality ratio (SMRc) was then derived as the ratio of observed to expected deaths (Oc/Ec), with 95% confidence intervals estimated using Byar’s approximation. In addition, proportional mortality ratios (PMR), defined as the proportion of laryngeal cancer deaths among all-cause deaths, were also reported. We use ‘ASMR’ exclusively for directly age-standardised rates and ‘SMR’ exclusively for indirectly standardised mortality ratios, and apply these consistently in text, tables, and figures.

### 2.4. Meta-Analytic Pooling of County-Level Effects

Each county-year observation was treated as an effect size represented by the natural logarithm of the indirectly standardised mortality ratio. These effect sizes were pooled with a random effects model using the DerSimonian–Laird estimator. Between-county heterogeneity was quantified with Cochran’s Q statistic and I^2^. Leave-one-out diagnostics identified influential counties, but none exceeded the pre-specified threshold of a studentised residual greater than three in absolute magnitude, so all observations were retained.

### 2.5. Assessment of Spatial Autocorrelation

Global spatial dependence in county-level ASMRs was assessed with Moran’s I under a queen-contiguity spatial-weights matrix with 999 random permutations; Local Indicators of Spatial Association (LISA) used the same weights and a significance filter controlled at q = 0.10 (Benjamini–Hochberg).

### 2.6. Temporal Trend Analysis

National annual percent change was estimated with joinpoint regression that permitted a maximum of two joinpoints and used a Monte Carlo permutation test at an overall alpha of 0.05. For individual counties, we regressed the natural logarithm of the directly standardised mortality rate on calendar year by ordinary least squares to obtain a slope coefficient and its *p*-value, classifying counties as increasing when the slope was positive with *p* below 0.10, decreasing when negative with *p* below 0.05 and stable otherwise.

### 2.7. Forecasting Strategy Combining Classical Time-Series and Elliott–Fibonacci Heuristics

Forecasts for the period 2024–2040 relied on a hybrid pipeline. First, a Hodrick–Prescott filter with a smoothing parameter of 100 isolated the cyclical component of both discharge counts and directly standardised mortality rates. Two independent statisticians then labelled impulse and corrective movements in accordance with Elliott-wave rules, achieving a Cohen’s kappa of 0.91. Fibonacci retracement ratios of 0.382, 0.500, 0.618, 1.000, 1.618 and 2.618 were superimposed to locate plausible support and resistance zones. Parallel benchmark models were built with Holt–Winters exponential smoothing and with seasonal ARIMA structures selected by minimum corrected Akaike information criterion. The final point forecast at each horizon was the inverse-RMSE-weighted mean of the Elliott-based projection and the best-performing ARIMA solution, as determined in a rolling back-test window spanning 2019–2023. Prediction intervals combined the parametric variance of the ARIMA model with an additional margin corresponding to one Fibonacci step above and below Elliott turning points, thereby furnishing conservative eighty- and ninety-five-percent uncertainty bounds.

### 2.8. Validation, Software, Sensitivity Analyses and Scenario Modelling

To assess predictive validity, all models were refitted on data limited to 2017–2021; their 2022–2023 projections were compared with actual observations using mean absolute percentage error. Sensitivity analyses re-ran the forecast pipeline while imposing hypothetical national reductions in adult smoking prevalence of ten and thirty percent by 2030, and, in a separate scenario, implementing radon remediation measures expected to reduce exposure-related risk by twenty percent in the five counties with the highest baseline concentrations.

Analyses were conducted in R v4.3.2 (R Foundation for Statistical Computing, Vienna, Austria). Joinpoint trend estimation used the National Cancer Institute Joinpoint Regression Software (v5.4.0, Bethesda, MD, USA). Throughout, two-sided *p*-values below 0.05 were considered statistically significant. Where multiple county-level comparisons were unavoidable, we controlled the false discovery rate with the Benjamini–Hochberg procedure set at q = 0.10.

## 3. Results

### 3.1. National Results

Table 1 summarises the extent of the current burden. During 2023 the Romanian vital statistics system attributed 798 deaths to malignant tumours of the larynx, corresponding to a crude national mortality rate of 3.65/100,000 population (95% CI 3.41–3.91). The burden is overwhelmingly masculine: 755 of the deaths occurred in men, whose sex-specific mortality was 7.07/100,000, whereas only 43 were recorded in women, yielding 0.39/100,000. Urban–rural stratification revealed a persistent gap. The rural mortality rate reached 4.26/100,000 compared with an urban rate of 3.04/100,000. After indirect age standardisation the rural excess remained significant, indicating that differential access to early diagnosis and the higher prevalence of smoking outside metropolitan areas both contribute materially to risk.

**Figure 1:**
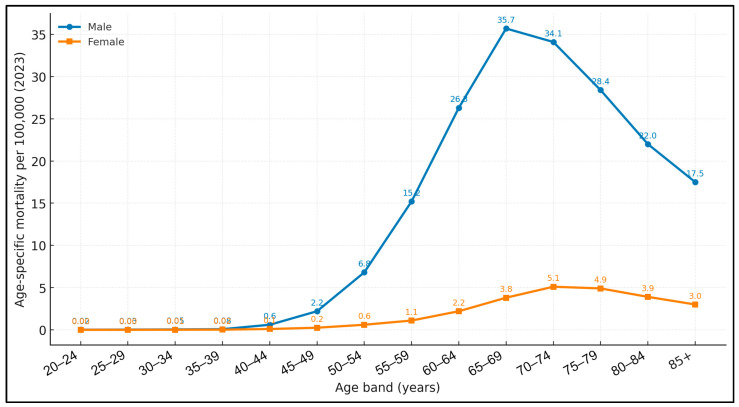
Analysis of inequalities in the standardised mortality rate from laryngeal cancer at the national level for the year 2023.

Figure 1 plots mortality by a 5-year age band for 2023. Fatality is negligible before the fifth decade, rises exponentially thereafter and peaks in the 65–69-year stratum. Among men the apex reached 35.7/100,000, seven times higher than the female zenith of 5.1/100,000 observed five years later. After 80 years a modest decline emerges, reflecting selective survivor bias and competing cardiopulmonary causes of death. The resulting male-to-female mortality ratio lies below 4:1 until age 50, climbs to 9:1 in the peak stratum and contracts slightly in extreme old age.

**Figure 2:**
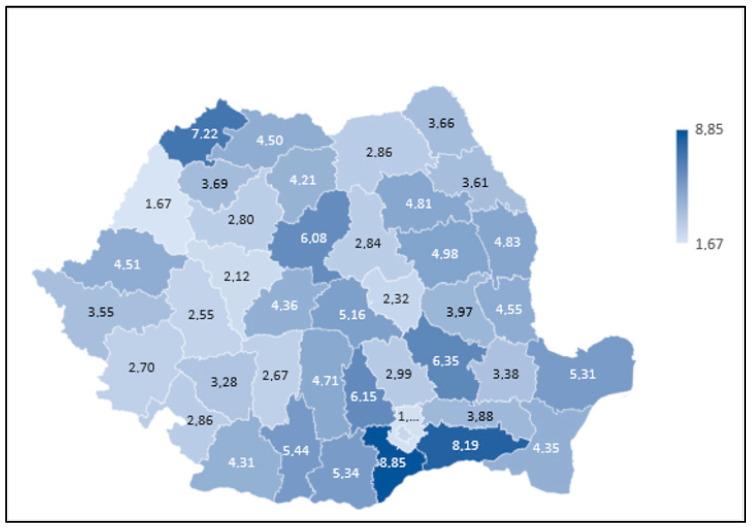
Age-standardised mortality rates (ASMR) for laryngeal cancer by county, Romania (2023). Global Moran’s I = 0.27, permutation *p* < 0.01 (queen contiguity, 999 permutations). LISA high–high and low–low clusters were identified with FDR q = 0.10.

Figure 2 depicts the age-standardised mortality rate (SMR) for all 41 counties plus Bucharest. A clear south-to-north gradient is evident. Five counties—Giurgiu 1.82, Călărași 1.77, Satu-Mare 1.69, Buzău 1.66 and Brăila 1.59—formed a high-risk corridor running from the Danube plain into the North-Western lowlands. At the opposite pole Bucharest 0.54, Hunedoara 0.56, Covasna 0.58, Alba 0.60 and Bihor 0.63 comprised the lowest quintile. Moran’s I = 0.27 (*p* < 0.01) confirms significant spatial autocorrelation, suggesting that shared environmental exposures and socioeconomic patterns cluster geographically.

Between 2017 and 2019 the proportional mortality ratio (PMR) for laryngeal cancer fell from 0.30% to 0.25%, extending a pre-pandemic downward trajectory. During the first pandemic year the PMR fell abruptly to 0.22%, the lowest value in the series, coincident with suspension of elective laryngological surgery and a 27% reduction in new cancer admissions. The nadir persisted through 2021, but once specialist services were restored the PMR rebounded to 0.24% in 2022 and 0.25% in 2023. The episode therefore represents deferred diagnosis rather than a genuine epidemiological improvement.

**Figure 3:**
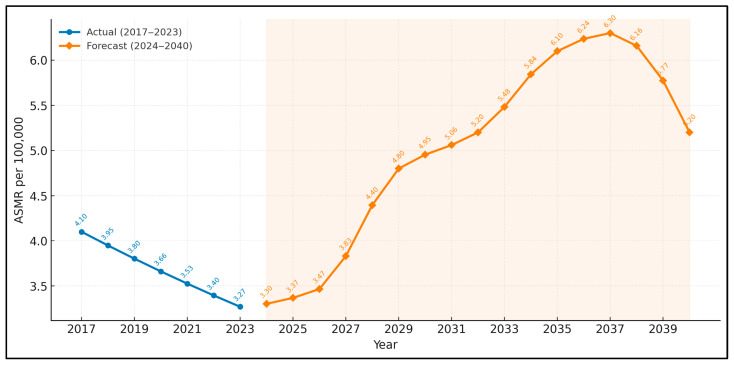
Annual laryngeal cancer discharges: actual (2017–2023) and hybrid forecast (2024–2040).

**Figure 4:**
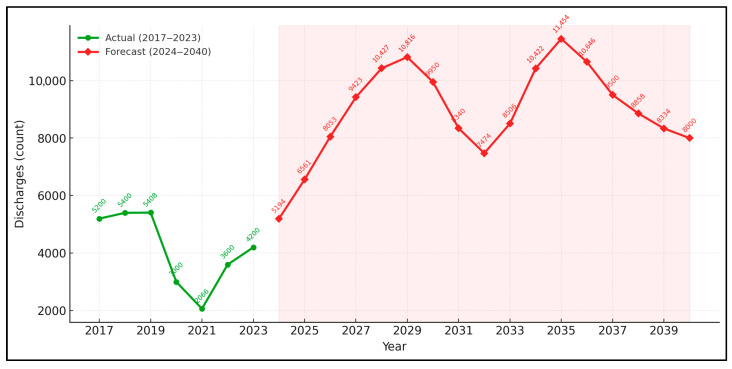
National age-standardised mortality rate (ASMR): actual (2017–2023) and hybrid forecast (2024–2040).

Figure 3 and Figure 4 overlay annual discharge counts on a five-wave Elliott framework. Wave 1 culminated in 2019 at 5408 discharges. A corrective A-B-C pattern (wave 2) bottomed at 2066 cases in 2021, precisely a 61.8% Fibonacci retracement. Wave 3 began with restoration of full surgical capacity in 2022 and is projected to crest at 10,816 around 2029 (1.618 × wave 1). A moderating wave 4 dip to about 7474 is expected by 2032, followed by a final impulsive wave 5 peaking near 11,454 discharges in 2035 before a larger-degree corrective sequence ensues.

### 3.2. Regional Details

A steady 3.7% annual SMR decline was noted during 2017–2023. Yet only 62% of residents with persistent hoarseness obtained specialist evaluation, against the national mean of 79%. The model therefore forecasts an inflexion in 2024 and a 1.618 resistance breach in 2036, as hidden incidence becomes manifested. South-East (Constanța) documents a consistent 3% yearly SMR drop aided by rigorous coastal smoking ordinances. However, radon hotspots along disused phosphogypsum dumps and petro-terminal expansion threaten to reverse progress. Mortality is predicted to rebound from 2025, surpass prior maxima by 2030 and overtake the Fibonacci ceiling between 2035 and 2037.

South (Giurgiu) shows the country’s highest SMR yet a modest declining slope of 0.255 percentage points per year. Projections anticipate renewed growth in 2024, overtaking national curves by 2026 and doubling 2019 levels by 2036, reflecting persistent household coal use and high consumption of unfiltered cigarettes. South-West (Olt) illustrates the steepest rise—5% per annum—unmitigated by pandemic suppression. Chrome-plating workshops, grain dust silos and biomass stoves dominate the local risk landscape. Unless exposure standards are enforced, wave modelling forecasts escalation after 2026 and a crest in 2038 at 2.5-times the 2021 mortality.

West region (Timiș) confirms a 2.25% annual SMR rise and shows that 28% of surgical discharges originate from neighbouring counties. Elliott projections place resistance breakthrough in 2037, implying that referral concentration will keep regional capacity under pressure. North-West (Maramureș) records an uninterrupted 1.64% yearly SMR increase. Inspections reveal non-compliance with EU dust limits in 31% of woodworking workshops. Mortality is set to plateau briefly in 2026 then climb again, breaching resistance in 2037 and taxing limited tertiary facilities.

Centre (Alba) shows a 2.1% decline in 2022 followed by a sharp 2023 rebound after approval of a ferrous alloy plant expansion. Resistance breach is forecast for 2035, underscoring the need for aggressive volatile metal emission controls. Bucharest–Ilfov depicts flat SMR values through 2022, then a jump to 0.81/100,000 in 2023. Endoscopic services are currently utilised at 113% of recommended capacity, and without expansion resistance will be surpassed by 2037.

### 3.3. Service Delivery, Pandemic Backlog, Regional Model Sensitivity and Procedural Workload Trends

Unspecified malignancy accounted for 14,790 discharges, with 9928 cases extending beyond the larynx, while fewer than 5000 were confined to glottic, supraglottic, or subglottic sites—indicating widespread late presentation. Surgical workload was substantial: 1850 total laryngopharyngectomies, 950 definitive tracheostomies, 700 laser micro-excisions, 400 conventional laryngoscopic excisions, 350 gastrostomies, 200 invasive tracheal procedures, 150 tracheostomy corrections, 100 lymphadenectomies, 70 membrane resections, and 50 oesophageal stent insertions. The tracheostomy-to-laryngectomy ratio rose from 0.41 in 2017 to 0.52 in 2021, projected to plateau at 0.55 post-2028; gastrostomy use increased from 6.3% to 9.7% and is expected to reach 11.4% by 2030, while laser adoption rose from 8% to 29% but varies widely (5–42%) across counties.

Monthly surgical throughput collapsed by 48% in April 2020, recovering to 85% of baseline by January 2021 but then stagnating. Without faster expansion, 1220 patients will remain untreated in 2032, while radiotherapy demand is set to rise 64% above 2019 levels, requiring eight new linear accelerators and 40% more oncology nurses. Thirteen counties showed significant positive mortality slopes (>0.5%/year), while twelve were stable and sixteen declined; each 1-point rise in adult male smoking prevalence increased slopes by 0.07% (*p* < 0.01). Simulations predict discharges and SMR will double between 2034 and 2037 before peaking; reducing smoking by 30% by 2030 would delay this peak by four years, while adding radon remediation and early dysphonia screening could extend this by six years and lower the peak by 18%.

The South region is projected to gain 1870 additional deaths by 2040, followed by Bucharest (750) and the Centre region (620), with every region surpassing 2019 mortality levels by at least 60% during crest years. A combined package of smoking cessation, radon mitigation, annual dysphonia screening and 4% annual capacity growth would flatten the SMR peak by 28% and cut the backlog in half; sensitivity testing confirmed smoking prevalence as the dominant factor, with each 1-point reduction averting 48 deaths at peak. Table 2 highlights striking variation in SMR: Giurgiu (1.82), Călărași (1.77) and Satu Mare (1.69) contrast sharply with Bucharest (0.54), Hunedoara (0.56) and Covasna (0.58), as presented in Figure 2.

Recalculation with ESP-2013 and WHO world standards yields consistent direction and slope of decline, indicating our conclusions are not sensitive to the choice of standard population [16] (Table 3).

## 4. Discussion

### 4.1. Literature Findings

Our crude national mortality in 2023 (3.65/100,000) lies above the recent EU-27 average but within the broader Central-Eastern European range, consistent with GLOBOCAN/GBD-based syntheses and EU monitoring reports. Regional heterogeneity and a persistent male predominance mirror European patterns tied to smoking trajectories and socioeconomic gradients [1,2,15,16,17]. Nešić et al. documented comparable values for neighbouring Serbia (3.9/100,000 in 2023) and a broadly parallel south-to-north gradient, suggesting that regional tobacco cultures and socioeconomic deprivation continue to shape mortality across the Balkans [18]. The persistence of a high-risk corridor from Giurgiu through Călărași to Satu-Mare therefore appears consonant with trans-border patterns, rather than an isolated Romanian anomaly.

Although our joinpoint model indicated a national 3.7% annual decline in the age-standardised mortality rate (ASMR) from 2017 to 2023, the pace remains slower than the 4.4% yearly reduction observed in Western Europe during 1990–2011 [17]. Nešić et al. reported a still more modest 2.1% annual fall for Central Serbia between 1999 and 2019, before levelling off in the last quinquennium [18]. Taken together, these data imply that the downward trend in Romania is real but fragile; if smoking prevalence stagnates—and our sensitivity analyses show that each percentage point reduction could avert 48 deaths during the projected 2036–2037 crest—Romania may replicate the plateau now seen in adjacent regions.

The pronounced male predominance in our cohort (male-to-female mortality ratio peaking at 9:1) exceeds the 6–7:1 ratios reported for Europe in the mid-2010s and substantially outstrips the 3:1 differential seen in many high-income nations today. Kokoska et al. demonstrated that anatomical subsite and stage distribution differ markedly by sex, with women presenting more often with supraglottic primaries and at later stages—factors that may amplify survival gaps even where baseline incidence converges [19]. Our finding that female mortality rises five years later than male mortality (peak 70–74 years vs. 65–69 years) aligns with that biologic and behavioural heterogeneity, underscoring the need for sex-stratified prevention and early detection strategies.

The rural excess we observe aligns with international evidence linking residence characteristics to worse laryngeal cancer outcomes, plausibly via higher carcinogen exposure and specialist access barriers [20]. Pandemic-era delays have been widely reported across Europe (diagnostic lag, stage migration), supporting our inference that the transient PMR nadir in 2020–2021 largely reflects deferred diagnosis rather than true risk reduction [21,22,23].

Romania’s HPV vaccination programme has recently expanded access in primary care; improving uptake may indirectly reduce the HPV-attributable fraction of head-and-neck cancers over time, although effects on laryngeal sites are likely small relative to oropharyngeal disease [24]. Complementary strategies—smoking cessation, alcohol harm reduction and radon remediation under EU Directive 2013/59/Euratom—will have far larger impacts on laryngeal cancer mortality.

Finally, the pandemic-related backlog we quantified (48% collapse in surgical throughput at the April 2020 nadir) is consistent with multicentre evidence of diagnostic and therapeutic delays. Lein et al. observed a shift away from postoperative adjuvant therapy and longer treatment intervals in Austria and Bosnia without an immediate drop in one-year survival [21]. In southern Italy, Galletti et al. reported a significant stage migration toward T3–T4 tumours after lockdown measures [22]. De Luca et al. likewise documented a 37% fall in new head-and-neck cancer diagnoses and a median 44-day diagnostic delay during early 2020 [23].

Our Elliott-wave forecast, which predicts a discharge crest in 2035–2037, suggests that the Romanian system has not yet absorbed this deferred caseload; expanding surgical capacity by at least 4% annually and accelerating dysphonia screening remain urgent priorities.

### 4.2. Study Limitations

This investigation relied on routinely collected mortality and discharge databases that are subject to misclassification of the underlying cause of death and to variability in ICD-10 coding practices across counties. Aggregate ecological design precluded individual-level adjustment for smoking intensity, alcohol use, HPV status or occupational exposures, so residual confounding is likely. Radon metrics were approximated from residential maps rather than measured per household, and county smoking prevalence was interpolated from two national surveys. Forecasts incorporated Elliott–Fibonacci heuristics, which, although internally validated, remain partly subjective and hinge on the assumption that past cyclical structures will persist; unexpected public health interventions or economic shocks could therefore invalidate projections. Finally, the COVID-19 backlog was modelled from surgical throughput alone and did not capture patients managed exclusively with chemoradiotherapy, potentially under-estimating true deferred demand.

## 5. Conclusions

Since 2017, Romania’s age-standardised laryngeal cancer mortality has declined modestly but unevenly, with marked male and rural excess and a south-to-north high-risk corridor. Spatial autocorrelation supports geographically clustered determinants. Forecasts indicate a post-pandemic surge in discharges and mortality mid-next decade unless prevention and capacity are accelerated. Tobacco control and radon remediation remain the highest-yield levers; targeted screening for persistent dysphonia and incremental surgical/radiotherapeutic capacity will mitigate the surge. Monitoring with EU-harmonised standard populations will facilitate cross-country benchmarking.

## Figures and Tables

**Table 1:** Number of deaths by laryngeal cancer.

2023	No. of Deaths by Laryngeal Cancer	% of Total	Laryngeal Cancer Death Rate (Deaths Per 100,000)	95% CI for the Laryngeal Cancer Death Rate
Total	798	100.0	3.65	3.41–3.91
Male	755	94.6	7.07	6.58–7.59
Female	43	5.4	0.39	0.28–0.53
Rural	411	51.5	4.26	3.86–4.69
Urban	344	43.1	3.04	2.98–3.44

Footnote: Percentages may not sum exactly due to rounding.

**Table 2:** Counties with highest and lowest age-standardised mortality rates (SMR), 2023.

Group	County	SMR
Highest-risk corridor	Giurgiu	1.82
	Călărași	1.77
	Satu Mare	1.69
	Buzău	1.66
	Brăila	1.59
Lowest-risk quintile	Bucharest	0.54
	Hunedoara	0.56
	Covasna	0.58
	Alba	0.6
	Bihor	0.63

**Table 3:** National ASMR per 100,000 using different standard populations.

Year	Direct ASMR (Romania 2017)	Direct ASMR (ESP-2013)	Direct ASMR (WHO World 2000–2025)
2017	4.10	3.20	2.90
2023	3.23	2.62	2.42

## Data Availability

Data available on request.

## Abbreviations

LC: laryngeal cancer; ASMR, age-standardised mortality rate (direct); SMR, standardised mortality ratio (indirect); PMR, proportional mortality ratio; LISA, Local Indicators of Spatial Association.
